# The efficacy of surgical treatment of cerebral arteriovenous malformations in a single academic institution: a case series

**DOI:** 10.3325/cmj.2021.62.353

**Published:** 2021-08

**Authors:** Martin Smrcka, Ondrej Navratil, Evzen Hovorka, Kamil Duris

**Affiliations:** 1 Masaryk University, Brno, Czech Republic; 2 Department of Neurosurgery, The University Hospital Brno and Faculty of Medicine, Masaryk University, Brno, Czech Republic; 3 Department of Pathological Physiology, Faculty of Medicine, Masaryk University, Brno, Czech Republic

## Abstract

**Aim:**

To report on patients who underwent surgical treatment of arteriovenous malformations (AVMs) at our institution.

**Methods:**

This retrospective single-center case series enrolled the patients who underwent surgical treatment of pial AVM at the Department of Neurosurgery, University Hospital Brno, between 2005 and 2019. The data are summarized as descriptive statistics presenting basic characteristics in all the patients and in sex or age subgroups.

**Results:**

Fifty patients were enrolled. The majority of AVMs were of Spetzler-Martin grade II (n = 27; 54%), localized supratentorialy (n = 43; 86%), and half of AVMs were ruptured. A total resection was performed in 48 patients (96%), and a good overall outcome was achieved in 44 patients (88%). Surgery-associated morbidity was 2%, and the mortality rate was 0% due to meticulous selection of patients for surgical treatment..

**Conclusion:**

Microsurgery is an appropriate method of treatment for S-M grade I-III pial AVMs. Microsurgery may be used to treat the majority of small-nidus AVMs with a low mortality and morbidity, when precisely planned and performed by an expert vascular team. The meticulous selection of patients for surgical treatment is crucial.

Cerebral arteriovenous malformations (AVMs) are congenital abnormalities of cerebral vessels, forming a nidus that directly shunts arterial blood to the venous system. The incidence of AVMs is around 1/100 000 persons per year (1). The symptoms include urgent medical conditions, such as intracerebral hemorrhage or subarachnoid hemorrhage, other medical conditions such as seizures or focal neurological deficit, as well as less severe conditions, such as headache (1). Rupture of AVMs is associated with a high morbidity (80%), and the mortality rate is 10%-30% (2). Permanent neurological deficit may be present in up to 42% of patients, while only 33% of patients survive bleeding without neurological deficit (3). The annual risk of AVM rupture is 2%-4% in the case of intact AVMs, and in the case of ruptured AVMs, the risk of re-rupture increases to 6%-8% during the first year and then slowly decreases (4,5).

Treatment of AVMs may be conservative or interventional. If possible, interventional treatment is preferred, because conservative treatment is associated with a risk of bleeding/rebleeding. The general goal of interventional treatment is to eliminate blood flow in AVM; only the complete elimination of blood flow may be considered as a curative intervention (6,7). The treatment strategy is always individualized, because every AVM and every patient is unique (8,9). The Spetzler-Martin grading system, originally published in 1986 and updated by Spetzler and Ponce in 2011, is a helpful tool for AVM treatment (10,11). Most neurosurgical centers use microsurgery for S-M grade I-II AVMs and the majority of S-M grade III AVMs, sometimes in combination with embolization or radiosurgery. Some of S-M grade III AVMs and the majority of S-M grade IV AVMs are treated with endovascular treatment or radiosurgery. Conservative treatment is used for some S-M grade IV AVMs and mainly for S-M grade V AVMs, which are predominantly managed with radiosurgery or conservative treatment.

Endovascular and radiosurgical treatment may be used separately, together, or in combination with surgical treatment. Endovascular embolization, using coils or Onyx, may be used for nidus reduction and sometimes for complete obliteration (12). Complete obliteration of AVMs may be achieved in approximately 13% of patients, but endovascular procedure is associated with complications in 7% of cases (13). Radiosurgery, using gamma knife or linear accelerator, is an option for AVMs that cannot be treated by surgery and that are not accessible with endovascular technique. Radiosurgery is beneficial for the treatment of small and deep AVMs, especially in older patients. The major disadvantage of radiosurgery is the latency before AVM obliteration, which usually takes 2-3 years (2). Complete obliteration may be achieved in 50%-90% of cases and it inversely correlates with nidus size (2). Radiosurgery is associated with approximately 5% risk of severe neurological complications or death (13).

Microsurgery is considered as a gold standard for AVMs treatment. A small nidus (up to 3 cm) can be completely removed in more than 94% of patients, with a good overall outcome in more than 90% cases (5). Surgical treatment of bigger AVMs or AVMs in eloquent areas is more complicated and thus less successful. Total excision of S-M grade IV AVMs is possible in 22% of cases and of S-M grade V AVMs in 17% of cases (5). This is why microsurgery is not usually used for the treatment of S-M grade IV and V AVMs. The general principle of surgical treatment is a resection of arterial feeders, followed by a nidus resection and a resection of draining veins. Modern methods, like neuronavigation or indocyanine- green videoangiography, help to improve the results of surgical treatment (2,14). Multimodal treatment combining microsurgery, endovascular treatment, and radiosurgery is the next step in the complex treatment of AVMs.

The Department of Neurosurgery University Hospital Brno has valuable experience in the treatment of AVMs. The aim of this article is to report on patients who underwent surgery for pial AVMs in our department during the past 15 years.

## Patients and methods

The study was approved by the Ethics Committee of University Hospital Brno (13-170221/EK). This retrospective, single-center case series enrolled patients who underwent surgical treatment of pial AVM at the Department of Neurosurgery, University Hospital Brno, between 2005 and 2019. Data were collected from medical documentation, and all the analyses were performed on anonymized data. The diagnosis of AVM was based on digital substraction angiography (DSA)/MRI angiography, which was repeated approximately one week after surgery to confirm the effect of surgical treatment. The following clinical characteristics were evaluated: clinical symptoms, localization and lateralization of AVM, S-M grade, 3-month outcome based on Glasgow Outcome Score (Good – GOS 5; Moderate – GOS 4, Severe – GOS 3). Data are presented as descriptive statistics in the whole sample and in sex or age subgroups. The results are presented in absolute (n) and relative (%) values and descriptive statistics (mean ± standard deviation) computed using GraphPad Prism version 5.00 for Windows (GraphPad Software, San Diego, CA, USA).

## Results

### Patient characteristics

The study enrolled 50 patients who underwent surgery for pial AVMs (28 men [56%]). The youngest patient was 13, the oldest 70 years old. The mean age was 39.6 ± 13.85 in the whole sample, 40.18 ± 13.24 in men, and 38.86 ± 14.88 in women. Twenty-eight (56%) patients were younger than 40 (up to 39, 40- group) and 22 (44%) patients were older than 40 (40 and older, 40+ group). There were 16 (32%) men and 12 (24%) women in the 40- group and 12 (24%) men and 10 (20%) women in the 40+ group.

### Symptoms and basic characteristics of AVMs

The most frequent symptom was bleeding, which was present in 50% of patients. Bleeding was more frequent in women (55% vs 46% in men) and older patients (64% vs 39% in young patients). Epilepsy was present in 34% of patients, slightly more often in men (36% vs 32% in women) and younger patients (43% vs 22% in older patients). Cephalea was a minor symptom (10% of patients), and 6% of AVMs were diagnosed accidentally.

The majority of AVMs (86%) were localized in the supratentorial region, with equal frequency in men (86%), women (86%), 40- (86%), and 40+ (86%) groups. Supratentorial AVMs were most frequent in the frontal and temporal lobes (Table 1). Finally, all the infratentorial AVMs (14%) were localized in the cerebellum.

**Table 1:** The basic characteristics of all the patients (All), men (M), women (F), patients younger than 40 (40-), and patients older than 40 (40+)*

	All		M		F		40-		40+	
	n	%	n	%	n	%	n	%	n	%
**Symptoms**										
**accidental**	3	6	3	11	0	0	2	7	1	5
**cephalea**	5	10	2	7	3	13	3	11	2	9
**epilepsy**	17	34	10	36	7	32	12	43	5	22
**bleeding**	25	50	13	46	12	55	11	39	14	64
**Localization**										
**Supratentorial**										
**F**	11	22	5	18	6	27	6	21	5	23
**F-P**	4	8	4	14	0	0	2	7	2	9
**F-T**	4	8	1	4	3	14	3	11	1	5
**F-T-P**	1	2	0	0	1	5	1	4	0	0
**O**	2	4	0	0	2	9	0	0	2	9
**P**	1	2	1	4	0	0	0	0	1	5
**P-O**	5	10	3	11	2	9	3	11	2	9
**T**	10	20	7	25	3	14	6	21	4	18
**T-O**	4	8	2	7	2	9	2	7	2	9
**T-P**	1	2	1	4	0	0	1	4	0	0
**total**	43	86	24	86	19	86	24	86	19	86
**Infratentorial**										
**cerebellum**	7	14	4	14	3	14	4	14	3	14
**Lateralization**										
**left**	19	44	11	46	8	42	12	50	7	37
**right**	19	44	11	46	8	42	9	38	10	53
**bilateral**	5	12	2	8	3	16	3	12	2	11
Spetzler-Martin **grade**										
**I**	15	30	6	21	9	41	6	24	6	27
**II**	27	54	19	68	8	36	15	60	12	55
**III**	7	14	3	11	4	18	3	12	4	18
**IV**	1	2	0	0	1	5	1	4	0	0
**V**	0	0	0	0	0	0	0	0	0	0
**Outcome**										
**good**	44	88	25	89	19	86	23	92	18	82
**moderate**	5	10	3	11	2	9	2	8	3	14
**severe**	1	2	0	0	1	5	0	0	1	4

*Abbreviations: F – frontal; P – parietal; O – occipital; T – temporal.

Lateralization of AVMs was uniform in the whole sample, 44% of AVMs were localized in the left hemisphere, 44% in the right hemisphere, and the remaining 12% had bilateral localization. Similar distribution were present in the male and female subgroup (46% left, 46% right). Lateralization to the right hemisphere was more frequent in the group 40- (50% right vs 38% left), while the distribution in group 40+ had an opposite trend (53% left vs 37% right)

According to the S-M scale, the majority of AVMs were grade II (54%), followed by grade I (30%) and grade III (14%). Similar distribution was observed in men (G II – 68%, G I – 21%, G III – 11%), in the group 40- (G II – 60%, G I – 24%, G III – 12%, G IV – 4%), and in the group 40+ (G II – 55%, G I – 27%, G III – 18%). The distribution in women was slightly different (G I – 41%, G II – 36%, G III – 18%, G IV – 4%) (Table 1).

### Treatment

**Figure 1:**
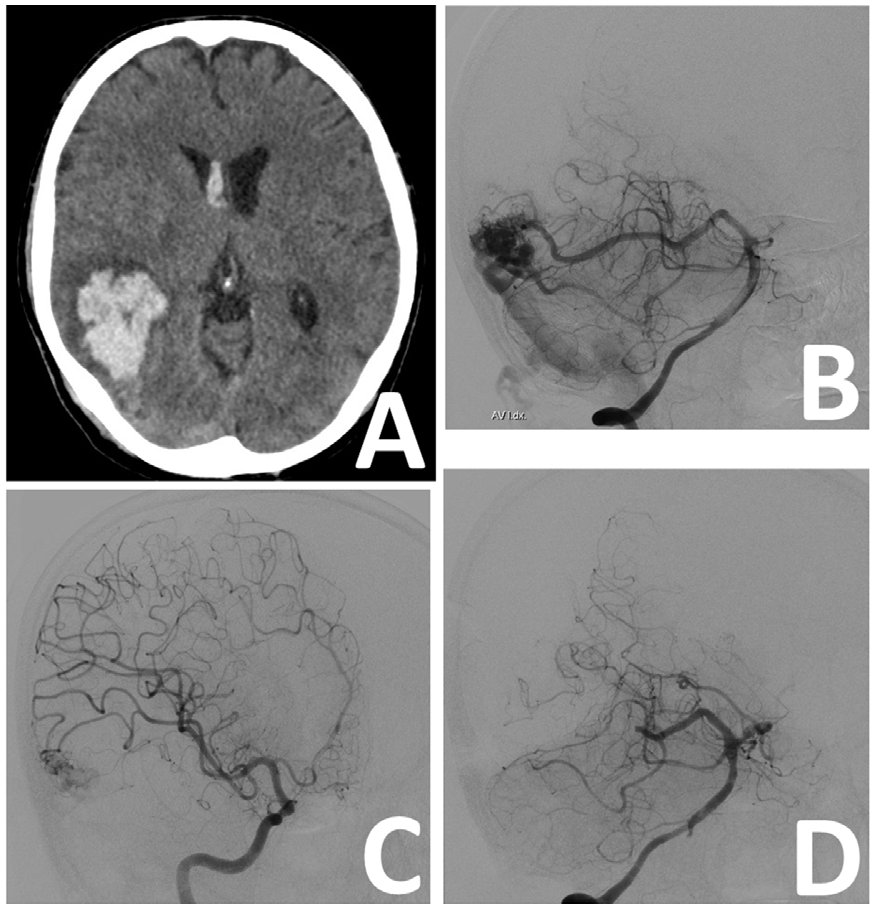
An illustrative case of a typical patient who underwent surgical treatment of arteriovenous malformations (AVM). A 44-year-old man with a history of sudden severe headache and nausea was referred by his general practitioner to the regional hospital. Computed tomography (CT) and CT angiogram revealed right temporo-occipital intracerebral hematoma caused by the AVM, Spetzler Martin grade II (**A**). The patient was admitted to the neurosurgical ICU and was neurologically intact. Angiography confirmed temporo-occipital AVM, fed mainly by posterior cerebral artery (**B**) on the right and terminal branches of the middle cerebral artery (**C**). There was also very subtle filling of the AVM from the occipital artery. Because of stable and intact neurological condition, endovascular preoperative embolization of the AVM was performed two weeks after the bleeding with almost total occlusion of the AVM after the intervention. The following day, the AVM was removed microsurgically, through navigated craniotomy and excision. On the fourth postoperative day, angiography showed total removal of the AVM without any filling (**D**). The course of the inpatient stay was uneventful, and the patient remained neurologically intact.

An illustrative case demonstrating typical patient in our cohort is shown in Figure 1. A total resection was performed in 48 patients (96%). Surgical resection was preceded by endovascular obliteration in two patients. In both patients, AVM was supplied by a single feeding artery arising from the arteria cerebri posterior. One patient (2%) underwent subtotal resection and in another patient AVM was not found during surgery (2%). This was a 55-year-old male patient with a small, S-M grade II AVM in the left central region. The surgical technique was careful because of localization in the motor area and the AVM was not found. Postoperative MRI was negative, no other intervention was performed, and the patient’s outcome at the moment of writing was good.

Forty-six patients (92%) had a newly diagnosed AVM, and the surgical treatment in our department was the first intervention, while four patients (8%) had treatment of AVM in medical history. Two of them underwent subtotal resection and the other two gamma-knife radiosurgery, which was not fully successful. Rebleeding was reported in one case and no response to radiosurgery in another case. All four patients underwent total resection in our department with a good outcome.

### Outcome

The 3-month outcome was good in 44 patients (88%), moderate disability was present in 5 patients (10%), and severe disability in 1 patient (2%). Similar distribution was present in all the subgroups: men (good – 89%, moderate – 11%), women (good – 86%, moderate – 9%, severe – 5%), the 40- group (good – 92%, moderate – 8%), and the 40+ group (good – 82%, moderate – 24%, severe – 4%). S-M grade I AVMs had a good outcome in 100% of the cases, S-M grade II in 85%, and S-M grade II in 71%. Moderate outcome was associated with the opposite trend (S-M I: 0%, S-M II: 11%, S-M III: 29%) (Table 2). Moderate disability was present in 5 patients (10%); disability was associated with initial bleeding in 4 patients and with a combination of initial bleeding and postoperative hematoma in 1 patient. Severe disability was related to initial bleeding in one case –a 70-year-old woman with S-M grade II AVM in the cerebellum experienced paraparesis. In summary, the morbidity rate in our sample was 10%, while the mortality rate was 0%.

**Table 2:** Patients' outcomes according to the Spetzler-Martin (S-M) grade

	S-M grade									
	I		II		III		IV		V	
Outcome	n	%	n	%	n	%	n	%	n	%
**Good**	15	100	23	85	5	71	1	100	0	0
**Moderate**	0	0	3	11	2	29	0	0	0	0
**Severe**	0	0	1	4	0	0	0	0	0	0

## Discussion

In this article we summarized our experiences with surgical treatment of AVMs and reported on 50 patients who underwent surgical treatment of pial AVM in our department during past 15 years.

AVMs are congenital pathologies, which are most often diagnosed in the fourth decade of life (15). Our findings are consistent with these observations – the mean age of our patients was 39.6 years. AVMs do not have gender predilection (15). This is in accordance with different gender dominance observed in various studies (13,15). In our study, there were slightly more women.

The most frequent clinical presentation of AVM was bleeding (50%), which is in accordance with the usually reported range of 45%-65% (16,17). The dominant features of AVMs in our study were supratentorial localization (86%) and S-M grade II (54%). This is similar to previous reports, which found supratentorial localization in 85% of patients and the S-M grades equal or below III as dominant features in patients who underwent surgical treatment (13,16).

A total resection was performed in 96% and a good outcome was achieved in 88% of patients. The overall morbidity rate was 10% and it was predominantly associated with initial bleeding. Surgery-associated morbidity was 2% and the mortality rate was 0%, which may be explained by the meticulous selection of patients for surgical treatment. These results are similar to previous reports, supporting the general opinion that microsurgery may be used to treat the majority of small-nidus AVMs with a low mortality and morbidity, when precisely planned and performed by an expert vascular team (2,5,13).

A critical step in the medical management of AVMs is the selection of treatment modality, which is always individualized according to the patient's overall status and AVM characteristics. The limiting factors of both surgical and endovascular treatments are severity of neurological symptoms, biological age, and comorbidities. The most important characteristics of AVM, which have to be taken into account when selecting treatment modality, are S-M grade, blood flow through AVM, AVM-related aneurysms, intracerebral hematoma, and hydrocephalus. Other crucial factors include the number and accessibility of arterial feeders, number of draining veins and their location (deep or superficial), and the density and compactness of the nidus. If the AVM is supplied by one or a couple of feeding arteries, obliteration before surgery is beneficial, making surgery easier and safer.

Microsurgery is a first-line treatment for S-M grade I-II AVMs and it is regularly used for the treatment of S-M grade III AVMs, often in combination with other treatment modalities. Preoperative planning is a crucial part of surgery. Basic characteristics of AVM, including localization, character of the nidus, its filling, arterial feeders, draining veins, and relation to eloquent areas, are assessed based on DSA and MRI imaging. Important aspects of surgery are the position of the patient with the head above the heart, larger craniotomy, and careful opening of the dura. The first step of AVM resection is an identification of feeders, both superficial and deep, and identification of draining veins, which must stay intact in the initial phase. Intra-arterial injection of indocyanine green dye may be very helpful to distinguish feeders from normal arteries and to visualize the dynamics of blood flow (18). Proximal control of arterial feeders, using temporary clips, precedes their resection. After the resection of arterial feeders, nidus resection is performed. The final step of AVM resection is coagulation and an interruption of draining veins. Meticulous hemostasis is essential because a problem with hemostasis may signalize residual AVM left behind. In the postoperative phase, normotension and normovolemia therapy is used. Analgesia, sedation, and antiepileptic drug are used individually.

The limitation of our study is that we did not use supplementary S-M grading system, which may provide additional information compared with traditional S-M grading system (19,20). Several studies have reported better predictive value of the supplementary grading system for estimating neurological outcome after the treatment (20,21).

Although we have a relatively clear idea about the treatment of AVMs, the final conclusion has to be based on future research. Randomized multi-center clinical trials are needed to determine the optimal AVM treatment modality. Recently, ARUBA trial has tried to answer the question of optimal AVM treatment, and although the results are controversial, it has significantly contributed to the field (22,23).

Microsurgery is an appropriate method of treatment for S-M grade I-III pial AVMs. A meticulous selection of patients for surgical treatment is crucial. Careful preoperative planning and individual approach are required. Precise technique, meticulous hemostasis, and accurate postoperative intensive care are essential for successful treatment. If all criteria are fulfilled, surgical treatment of S-M grade I-III AVMs is a safe and efficient modality, which is associated with a low morbidity and mortality.
